# Probiotic Potential of *Lactobacillus* Strains Isolated From Fermented Vegetables in Shaanxi, China

**DOI:** 10.3389/fmicb.2021.774903

**Published:** 2022-02-01

**Authors:** Chen Liu, Wen-jiao Xue, Hao Ding, Chao An, Sai-jian Ma, Yao Liu

**Affiliations:** Shaanxi Institute of Microbiology, Xi’an, China

**Keywords:** probiotic, *in vitro*, *Lactobacillus*, handmade fermented vegetables, Shaanxi

## Abstract

The objective of this study was to assess *in vitro* probiotic potential of *Lactobacillus* strains derived from artisanal fermented vegetables in Shaanxi, China. In total, 74 acid-producing Gram-positive strains with rod-shaped under the microscope were isolated from 16 samples of spontaneously fermented vegetables. Out of 74 strains, 26 showed high survival rate under low pH and high bile salts conditions and were subjected to molecular identification by 16S rRNA gene sequencing analysis. The results showed that 15 isolates belonged to *Lactobacillus plantarum*, 9 isolates belonged to *Lactobacillus brevis*, and the 2 remaining strains belonged to *Weissella viridescens*. The 24 *Lactobacillus* strains were investigated for their survival rate to transit simulated gastrointestinal tract, cell surface hydrophobicity, auto-aggregation, co-aggregation with pathogen, adhesion to Caco-2, antimicrobial activity, antibiotics susceptibility, radical scavenging ability, α-glucosidase inhibition, and the cholesterol assimilation. The results showed that the probiotic characteristics were strain-dependent, and several strains exhibited great probiotic potential with specific health benefits, which indicated that they might be excellent candidates for production of functional foods. Interestingly, it was first found that *L. plantarum* generally had higher antibacterial activities, α-glucosidase inhibition ability, and antibiotics susceptibility compared to *L. brevis* in this study. The results indicated that *Lactobacillus* strains isolated from fermented vegetables in Shaanxi, China, could be exploited as a promising novel probiotic source.

## Introduction

While consumption of dairy products has drawbacks, including lactose intolerance and high cholesterol content, non-dairy probiotic food products have received increasing attention because of their unique characteristics, such as increasing nutritional value (Sarao and Arora, 2015; Min et al., 2019). However, the incorporation of probiotic bacteria into non-dairy products encountered a few challenges due to limited access to probiotic strains, especially for small manufacturers (Zielinska et al., 2015; Min et al., 2019). Therefore, there is a high demand to select new bacteria strains with different functional properties for commercial production of novel probiotic foods and for improving quality of traditional fermented food.

According to FAO/WHO Report (FAO/WHO, 2002), probiotics are defined as “live microorganisms which when administered in adequate amounts confer a health benefit on the host.” To benefit human health, probiotics must survive after passing through the gastrointestinal tract (Ramos et al., 2013). Thus, it is essential for the bacteria to have abilities to withstand low pH in the stomach, digestive enzymes, and bile salts of the small intestine (Sarao and Arora, 2015; Tokatli et al., 2015; Zielinska et al., 2015). Meanwhile, the colonization ability in the intestinal wall is considered as a desirable property of probiotic bacteria (Nami et al., 2019b). Hydrophobicity and auto-aggregation could contribute to the colonization ability of probiotics in the intestinal wall while co-aggregation represents a defensive barrier for the colonization of pathogenic microorganisms (Choi et al., 2018; Nami et al., 2019b). Other functional properties used to characterize probiotics include antagonistic activities (Choi et al., 2018; Jia et al., 2021), α-glucosidase inhibition activity (Chen et al., 2014), and antioxidant activity (Cao et al., 2019; Ragul et al., 2020). In addition, the safety assessment is essential before a strain is used as a probiotic in the food industry (FAO/WHO, 2002; Nami et al., 2019b).

Lactic acid bacteria (LAB), particularly *Lactobacillus* genera, is the most widely used probiotic due to their “generally recognized as safe” (GRAS) status and their potential health-promoting effects. Fermented foods are proved to be rich sources for new LAB strains with excellent probiotic characteristics (Yang et al., 2017; Cao et al., 2019; Li et al., 2019; Yi et al., 2020; Zheng et al., 2020; Jia et al., 2021; Wu et al., 2021). Fermented vegetables have a long history in many countries and LAB is the most important fermenting microorganism they contain (Zhang et al., 2018; Liu et al., 2019). Although fermented fruit and vegetable products have been used as raw materials for probiotic microorganisms in several studies (Tokatli et al., 2015; Zielinska et al., 2015; Monika et al., 2017; Choi et al., 2018; Chen et al., 2019; Jia et al., 2021), there are still inadequate investigations for probiotic potential of artisanal fermented vegetables especially in Northwest China, where fermented vegetables have a long history of thousands of years. It was reported that the diversified fermented vegetables in Northwest China harbored rich sources of LAB strains (Zhang et al., 2018; Liu et al., 2019), which could be exploited as a promising source for novel probiotics.

The objective of this study was to assess probiotic potentials of *Lactobacillus* strains isolated from handmade fermented vegetables in Shaanxi, China. Those isolates with high bile tolerance and low pH tolerance were investigated for their survival rate to transit simulated gastrointestinal tract, cell surface hydrophobicity, auto-aggregation, co-aggregation with pathogen, adhesion to Caco-2, antimicrobial activity, antibiotics susceptibility, radical scavenging ability, α-glucosidase inhibition, and the cholesterol assimilation *in vitro*.

## Materials and Methods

### Isolation of Bacterial Strains

Samples of fermented food (pickled Chinese cabbage, pickled chili, pickled cabbage, pickled carrot, pickled leaf mustard, pickled cowpea, and pickled ginger) used for the isolation of LAB were purchased from different local markets in Shaanxi, China. Samples were put in sterilized tubes and transported to the laboratory at Shaanxi Institute of Microbiology (Xi’an, China) within 12 h. The isolation was performed using the method described before (Pringsulaka et al., 2012) with slight modification. The fermented food (1 g) was weighed, minced with sterile scissors, and was added to 10 mL sterile water and mixed thoroughly. Each sample was serially diluted with 0.85% (w/v) sterile saline solution and cultured on the de Man, Rogosa Sharpe (MRS) agar supplemented with 2% (w/v) CaCO_3_ at 37°C for 48 h. The colonies, which were round, milky, and had a clear halo surrounding them, were picked and purified by streaking on MRS plates. Cell morphology was examined by optical microscopy (100X, Olympus BX41, Japan) and Gram staining. The isolates were stored at –80°C in MRS broth containing glycerol (25%, v/v).

### Tolerance Against Low pH and Bile Salt

The strains were preliminarily selected by incubating them in MRS with and without 0.3% bile salts (0% as control) at an inoculum size of 1% (v/v). The strains were then incubated at 37°C for 4 h. The OD values at 600 nm were measured after 4 h cultivation with and without 0.3% bile salts, respectively. Bile tolerance was expressed as follows:


$$Suppression\left(\%\right)=\left(\frac{O\,D_{600}\,\left(0\%\,b\,i\,l\,e\,s\,a\,l\,t\right)-O\,D_{600}\,\left(0.3\%\,b\,i\,l\,e\,s\,a\,l\,t\right)}{O\,D_{600}\,\left(0\%\,b\,i\,l\,e\,s\,a\,l\,t\right)}\right)\times 100\%$$

The tolerance against low pH was determined on the strains with high bile salt tolerance. The strains were incubated in MRS with pH 2.0, pH 2.5, and pH 6.4 (as a control) for 24 h. The OD values at 600 nm were measured at 0 h and 24 h cultivation, respectively. The survival rate was calculated as follows:


$$Survival\left(\%\right)=\left(\frac{O\,D_{600}\,\left(24\,h\right)}{O\,D_{600}\,\left(0\,h\right)}\right)\times 100\%$$

### 16S rRNA Analysis

Molecular identification of selected strains was performed according to the method described before (Wang et al., 2020). Briefly, microbial genomic DNA was extracted by using TaKaRa Universal DNA extraction kit (Cat# 9765) (TaKaRa Bio Inc., Shiga, Japan). Bacterial 16S rRNA gene sequences were PCR-amplified from each sample using the 27F-1492R primers. PCR was carried out with an automated thermal cycler (Biometra Germany) using Taq polymerase (TaKaRa Bio, Inc., Shiga, Japan). Sequences were determined at BGI Biotechnology (Shenzheng, China) and analyzed using BLAST (NCBI, Bethesda, MD, United States). Sequences obtained in this study were submitted to the GenBank database with accession numbers from OK021606 to OK021632 and the neighbor-joining phylogenetic tree was constructed using MEGA 5.05.

### *In vitro* Resistance to Simulated Gastrointestinal Juices

The survival of selected LAB through simulated gastrointestinal juices was examined according to a previous report with slight modification (Jamyuang et al., 2019). After two generations of cultivation, the LAB strain was inoculated with 1% (v/v) inoculum in MRS liquid medium and incubated for 18 h at 37°C. Cells were harvested by centrifugation (8000 rpm at 4°C for 5 min) and cell number was adjusted to 10^9^ CFU/mL by adding PBS solution. The obtained bacterial suspensions (1 mL) were inoculated to 9 mL of filter sterilized simulated gastric juice solution [3 g/L pepsin (p7000, sigma), pH 2.0] at 37°C for 60 min. The gastric juice solution was removed by centrifugation (8000 rpm, 5 min) and subsequently re-suspended in 9 mL of filter sterilized simulated small intestinal juice solution [1 g/L trypsin (T105532, Aladdin), 0.3% bile salt, pH 8.0]. Samples were incubated statically at 37°C for 120 min. Then, the intestinal juice was removed by centrifugation (8000 rpm at 4°C for 5 min). The cell pellet was suspended in a sterile 0.85% NaCl (w/v) solution. Survival cells were counted on MRS plates. The survival rate (%) was calculated by the following equation:


$$Survival\left(\%\right)=\left(\frac{\log\left(N_{t}\right)}{\log\left(N_{0}\right)}\right)\times 100\%$$

where N_0_ and N_*t*_ represent viable bacterial cells before and after growth in the simulated gastrointestinal tract, respectively.

### Hydrophobicity

After overnight incubation, bacterial cultures were centrifuged at 8000 rpm for 5 min (4°C). The cell pellets were washed twice and re-suspended in PBS buffer (pH 7.20) to achieve OD_600_ value of approximately 0.4 (labeled as A_0_). The cell suspension (3 mL) was blended with 1 mL chloroform and the mixture was vortexed for 30 s. Then it was left at room temperature for 30 min to separate the aqueous and organic phases. The aqueous phase (1 mL) was removed carefully, and the absorbance (A_*X*_) was measured at 600 nm. The hydrophobicity was calculated according to following equation:


$$Hydrophobicity\left(\%\right)=\left(\frac{A_{0}-A_{X}}{A_{0}}\right)\times 100\%$$

### Auto-Aggregation and Co-aggregation Abilities

The aggregation abilities of selected LAB were evaluated in a previous study (Choi et al., 2018). Overnight cultured LAB strains were collected by centrifugation (8000 rpm, 4°C, 15 min). The cell pellets were washed twice and re-suspended to 10^8^ CFU/mL with PBS buffer (pH 7.2).

For the auto-aggregation assay, 4 mL LAB suspension was incubated at 37°C for 6 h without agitation. Then 0.5 mL of suspension was transferred to a new tube and mixed with 1.5 mL PBS buffer. The OD values at 600 nm were measured and the auto-aggregation rate was calculated as follows:


$$Auto-aggregation\left(\%\right)=\left(\frac{A_{0}-A_{t}}{A_{0}}\right)\times 100\%$$

where A_0_ and A_*t*_ represent the OD_600_ values after 0 and 6 h incubation, respectively.

For co-aggregation assay, *Shigella flexneri* CMCC51574, *Salmonella paratyphi B* CMCC50094, and *Escherichia coli* CMCC44102 were used as pathogenic strains. The LAB strains and the pathogenic strains liquid concentration were adjusted to 10^8^ CFU/mL as described above. Equal volumes (2 mL) of LAB strains and pathogenic strains were mixed and vortexed for 10 s followed by an incubation at 37°C for 4 h without shaking. Cell suspensions of each single strain were used as controls. Based on the method proposed by Handley et al. (1987), co-aggregation percentage was calculated as follows:


$$Co-aggregation\left(\%\right)=\left(\frac{\left(A_{L\,A\,B}+A_{p\,a\,t}\right)/2-A_{m\,i\,x}}{\left(A_{L\,A\,B}+A_{p\,a\,t}\right)/2}\right)\times 100\%$$

where*A*_*LAB*_, *A*_*pat*_, and *A*_*mix*_ represents the OD_600_ values of control tubes and mixture after 4 h incubation, respectively.

### Adhesion to Caco-2 Cells

The adhesion ability to Caco-2 cells was evaluated following the method described by Srimahaeak et al. (2021) with some modifications. The Caco-2 cells were cultured in DMEM-high glucose medium (HyClone, United States) supplemented with 10% (v/v) fetal bovine serum (FBS) (Bioexplorer, United States), 1% (v/v) nonessential amino acids solution (AAS) (Solarbio, China) and 1% (v/v) penicillin-streptomycin (Bioexplorer, United States) until they approached 80–90% confluence. The cells were trypsinized with 1% (w/v) of trypsin-EDTA (Bioexplorer, United States) solution to make new passages. For the adhesion assay, cells were seeded into 24-well cell culture plates and incubated at 37°C, 5% CO_2_ for 15 days in a humidified atmosphere to obtain cell differentiation. One milliliter of LAB suspension (1 × 10^8^ CFU/mL) in DMEM was added to each well of the 24-well cell culture plate and co-incubated with Caco-2 monolayers for 1 h at 37°C with 5% CO_2_. The wells were washed twice with a pre-warmed Dulbecco’s phosphate buffered saline (DPBS) (pH 7.2, without Ca and Mg) (Procell, China) to remove non-attached cells of LABs and adherent bacteria were detached with DPBS containing 1% (v/v) Triton X-100. The serially diluted lysates were then plated onto MRS agar to determine the number of adherent bacteria and the experiment was carried out in triplicate. Results were expressed as the percentage of bacteria adhered with respect to the number of bacteria added.

### Antimicrobial Activity

Well diffusion was performed to evaluate the inhibitory effects of LAB strains on the growth of indicator strains. In this study, *Shigella flexneri* CMCC51574, *Salmonella paratyphi B* CMCC50094, and *Escherichia coli* CMCC44102 were used as indicator bacteria. The indicator bacteria suspension (50 μL, approximately 10^8^ CFU/mL) was added to 200 mL MRS agar, mixed thoroughly, and poured plate. Eight-millimeter wells were punched on plates using sterile borer. Then, each well was filled by 80 μL of filtered supernatant and plates incubated overnight at 37°C. The inhibition zone was measured in millimeters around the well.

### Antibiotic Susceptibility

Antibiotic sensitivity was determined using the agar disk diffusion assay method based on a previous study (Nami et al., 2019b). Eight clinically important antibiotics were used to determine the antibiotic susceptibility of the isolated strains. The strains were incubated in MRS broth at 37°C for 18 h. Then, 50 μL of the suspension (approximately 10^8^ CFU/mL) was mixed with 200 mL MRS agar thoroughly and was poured on plate. Antibiotic disks were manually placed on the plates by using sterilized forceps. The diameter of inhibition (mm) around each disk was measured after overnight incubation at 37°C. The strains were grouped into sensitive, moderately sensitive, and resistant according to the Clinical Laboratory Standards Institute (CLSI) recommendations (CLSI, 2020).

### Preparation of Cell-Free Supernatants, Intact-Cells, and Intra-Cellular Cell Free Extracts

The LAB strains were cultivated in MRS broth at 37°C for 16 h∼18 h. The fermentation broth was centrifuged at 8000 rpm for 5 min under 4°C and the supernatants were collected and filtered through a Millex^®^ 0.22-μm filter (Millipore) to obtain cell-free supernatant (CFS). The harvested cell pellets were washed twice with 0.1 mM phosphate buffer solution (PBS, pH 7.4), re-suspended in PBS, and adjusted to 10^9^ CFU/mL. The re-suspended solutions were divided into two aliquots. One aliquot was used as intact cells (IC), and the other aliquot was ultra-sonicated at 800 W for 30 min (5 s sonication, 7 s interval) in an ice bath. The resulting supernatant was harvested and was filter sterilized to obtain intra-cellular cell-free extracts (CFE).

### Radical Scavenging Activity

The stable radical DPPH (2,2-Diphenyl-1-picrylhydrazyl) and the stable cation radical ABTS 2, 2’-Azino-bis (3-Ethylbenzothiazoline-6-sulfonic acid) were used to measure the free radical scavenging activity of LAB strains as reported previously (Cao et al., 2019; Ragul et al., 2020). Intact cells or CFE or CFS (1 mL) were mixed with 1 mL DPPH solution in methanol (200 μM) vigorously and kept at room temperature in the dark for 30 min. Trolox (150 μg/ml) was used as a positive control. The absorbance was measured at 517 nm and DPPH scavenging ability was calculated as:


$$DPPHscavengingeffect\left(\%\right)=\left[\frac{1-A_{517}\,\left(s\,a\,m\,p\,l\,e\right)}{A_{517}\,\left(b\,l\,a\,n\,k\right)}\right]\times 100\%$$

The solution of 7 mM ABTS^+^ was prepared in 2.45 mM potassium persulfate followed by incubation at room temperature for 12 h. The ABTS^+^ solution was diluted in deionized water to achieve OD_734_ value of approximately 0.7. Twenty microliters of intact cells, CFS, or CFE were vortexed with 2 mL of ABTS^+^ working solution for 30 s and incubated at room temperature for 6 min. Trolox (350 μg/ml) was used as a positive control. The absorbance was read at 734 nm and ABTS^+^ scavenging ability was defined as follows:


$$ABTS^{+}scavengingeffect\left(\%\right)=\left[\frac{1-A_{734}\,\left(s\,a\,m\,p\,l\,e\right)}{A_{734}\,\left(b\,l\,a\,n\,k\right)}\right]\times 100\%$$

### α-Glucosidase Inhibitory Activity of Lactic Acid Bacteria Strains

The α-glucosidase inhibition assay was performed following Chen’s method (Chen et al., 2014) with slight modification. Twenty-five microliter samples (CFS/IC/CFE) were added to 96-wells microplate containing 50 μL of 20 mM p-nitrophenyl α-D-glucopyranoside (pNPG) and 50 μL of 0.1 M PBS (pH 6.8). The samples were then incubated at 37°C for 10 min and then 30 μL of α-glucosidase solution (20 U/mL) (Solarbio, China) was added and incubated at 37°C for 20 min. The reaction was stopped by adding 50 μL of 1 M Na_2_CO_3_. p-nitrophenol released from pNPG was measured spectrophotometrically at 405 nm. The percent inhibition of α-glucosidase was calculated by the following equation:


$$Inhibitonpercentage\left(\%\right)=\left(1-\frac{A}{B}\right)\times 100\%$$

where A is the absorbance of the reactants with sample and B is the absorbance of blank group containing reactants without sample solution.

### Cholesterol Assimilation

MRS-CHOL broth was prepared by supplementing MRS broth with cholesterol at a concentration of 0.1 g/L. Specifically, cholesterol (0.1 g), bile salt (2.0 g), and sucrose octaacetate (0.1 g) were mixed with 1.0 mL of tween 80 and followed by adding 5 mL glacial acetic acid. The mixture was dissolved at 60°C and exposed to ultrasound of 130 W (2 s sonication, 3 s interval) for 30 min. The prepared MRS liquid medium was quickly added to the above mixture. The mixture was kept stirring until a homogeneous colloidal solution was formed.

After autoclaving, the MRS-CHOL broth was inoculated with 1% (v/v) bacterial culture (approximately 10^8^ CFU/mL) at 37°C for 24 h. Bacterial broth was centrifuged at 10,000 rpm for 15 min to remove cells. Cholesterol concentration was determined (Rudel and Morris, 1973; Nami et al., 2019b) and cholesterol removal percentage was calculated as follows:


$$Cholesterolremovalpercentage\left(\%\right)=\left(\frac{C_{0}-C_{t}}{C_{0}}\right)\times 100\%$$

where C_0_ is the concentration of cholesterol at the initial medium and C_*t*_ is the concentration of cholesterol at the end of inoculation.

### Statistical Analysis

All experiments were performed in triplicate. Means and standard deviations were calculated. Statistical analysis of data was carried out using SPSS (Ver. 19.0 SPSS, Chicago, IL, United States). The comparisons of means among different treatments were performed by one-way ANOVA at a significance level of *P* < 0.05.

## Results

### Prescreening and Identification of Isolates

**FIGURE 1:**
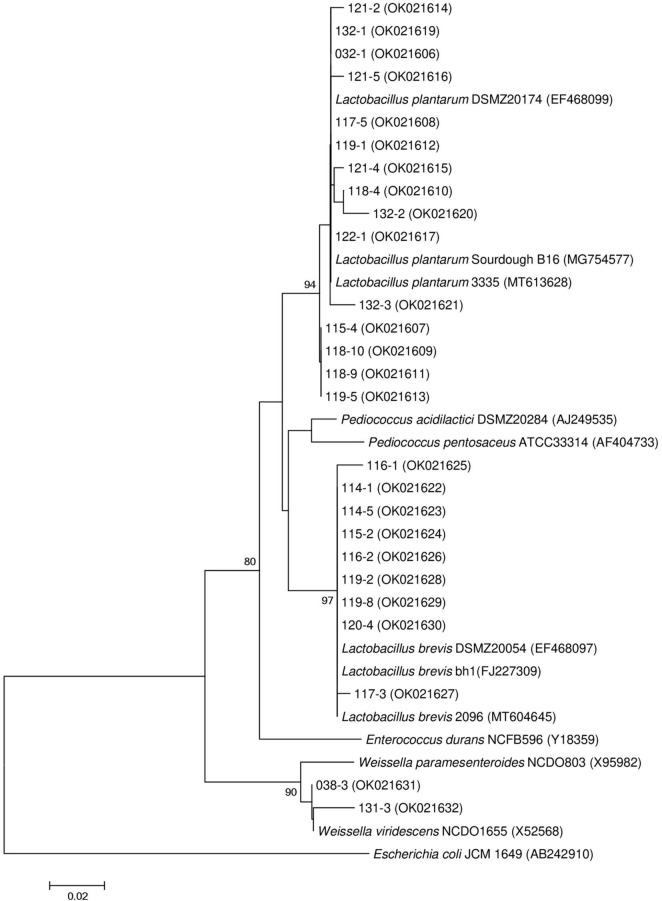
Phylogenetic tree of lactic acid bacteria based on neighbor-joining distance analysis of 16S rRNA gene sequences.

A total of 74 strains, Gram-positive, acid-producing, and rod-shaped under the microscope, were isolated from 16 samples of spontaneously fermented vegetables, including fermented cabbage (13 isolates), fermented carrot (14 isolates), fermented chili (4 isolates), fermented Chinese cabbage (32 isolates), fermented cowpea (4 isolates), fermented cucumber (2 isolates), fermented ginger (2 isolates), and fermented leaf mustard (3 isolates), in Shaanxi, China. All 74 strains were subjected to a bile tolerance prescreening (Supplementary Table 1) and 27 of them, which showed below 50% inhibition rate in MRS medium containing 0.3% bile for 4 h, were selected for pH tolerance test. The results showed 26 out of 27 LAB strains survival rate were higher than 100% at both pH 2.5 and pH 2.0 condition (Supplementary Table 2). The 26 isolates were identified based on 16S rRNA sequencing and the results are shown in Table 1. Phylogenetic tree of the 26 LAB strains is shown in Figure 1. All 16S rRNA gene sequences showed 98.05% ∼ 100% similarity with the *Lactobacillus* spp. Fifteen strains of *Lactobacillus plantarum* accounted for 57.69% of all strains that passed the initial screening, followed by *Lactobacillus brevis* with 9 strains (34.62%), and the remaining 2 strains belonged to *Weissella viridescens*, as shown in Table 1. Since the genus *Lactobacillus* was generally considered as GRAS (generally recognized as safe) (Min et al., 2019), we focused on the *Lactobacillus* strains for further study.

**TABLE 1:** Identification of lactic acid bacteria isolated from fermented vegetables in Shaanxi, China.

Strain	Origin	Identification (References)	GenBank Accession No.	Percentage similarity (%)
032-1	Pickled Chinese cabbage	Lactobacillus plantarum 3360	OK021606	100.00%
038-3	Pickled Chinese cabbage	Weissella viridescens 6996	OK021631	99.86%
114-1	Pickled chili	Lactobacillus brevis gp60	OK021622	99.32%
114-5	Pickled chili	Lactobacillus brevis 3676	OK021623	99.73%
115-2	Pickled cabbage	Lactobacillus brevis 2096	OK021624	99.12%
115-4	Pickled cabbage	Lactobacillus plantarum 6401	OK021607	99.85%
116-1	Pickled Chinese cabbage	Lactobacillus brevis gp71	OK021625	98.84%
116-2	Pickled Chinese cabbage	Lactobacillus brevis 4116	OK021626	99.93%
117-3	Pickled carrot	Lactobacillus brevis 1992	OK021627	98.05%
117-5	Pickled carrot	Lactobacillus plantarum 2008	OK021608	98.59%
118-10	Pickled Chinese cabbage	Lactobacillus plantarum 8m-21	OK021609	99.93%
118-4	Pickled Chinese cabbage	Lactobacillus plantarum 7071	OK021610	98.38%
118-9	Pickled Chinese cabbage	Lactobacillus plantarum 6093	OK021611	98.52%
119-1	Pickled Chinese cabbage	Lactobacillus plantarum Sourdough_B16	OK021612	99.73%
119-2	Pickled Chinese cabbage	Lactobacillus brevis 5644	OK021628	98.12%
119-5	Pickled Chinese cabbage	Lactobacillus plantarum JY28	OK021613	99.52%
119-8	Pickled Chinese cabbage	Lactobacillus brevis b4	OK021629	99.18%
120-4	Pickled leaf mustard	Lactobacillus brevis gp118	OK021630	98.78%
121-2	Pickled cowpea	Lactobacillus plantarum 3748	OK021614	99.05%
121-4	Pickled cowpea	Lactobacillus plantarum 3383	OK021615	98.71%
121-5	Pickled cowpea	Lactobacillus plantarum 3356	OK021616	100.00%
122-1	Pickled cabbage	Lactobacillus plantarum ZDY36a	OK021617	99.66%
131-3	Pickled ginger	Weissella viridescens 6996	OK021632	98.85%
132-1	Pickled carrot	Lactobacillus plantarum 5600	OK021619	99.11%
132-2	Pickled carrot	Lactobacillus plantarum 7232	OK021620	100.00%
132-3	Pickled carrot	Lactobacillus plantarum 5955	OK021621	98.52%

### *In vitro* Resistance to Simulated Gastrointestinal Juices

After sequential exposure to simulated gastrointestinal juices, survival rate of all test organisms remained above 68% and *L. plantarum* 115-4 had the highest survival rate of 96.70% (Table 2). Results showed that 20 out of 24 strains had a survival rate higher than 95% after 1 h incubation in simulated gastric juice and after the following 2 h incubation in simulated small intestinal juice there were 10 isolates with a survival rate higher than 90%. The results indicated that the test strains have better acid tolerance compared to the bile tolerance. The survival rate of *L. plantarum* ranged from 75.01 to 96.7% while *L. brevis*’s survival rate ranged from 68.17 to 96.6%. There was no significant difference between the two species in terms of tolerance to simulated gastrointestinal. The tolerance differences were strain-dependent rather than species-dependent, which was consistent with the low pH and high bile salts test results.

**TABLE 2:** *In vitro* resistance to simulated gastrointestinal juice of *Lactobacillus* strains from fermented vegetables in Shaanxi, China.

Species	Strains	Initial cell counts (log cfu/mL)	Simulated gastric juice (pH 2.0, 1 h)		Simulated intestinal juice (pH 8.0, 2 h)	
			Cell counts (log cfu/mL)	Survival rate (%)	Cell counts (log cfu/mL)	Survival rate (%)
*L. brevis*	114-1	8.24 ± 0.17	7.91 ± 0.04	95.96 ± 0.46^l^	6.04 ± 0.03	73.32 ± 0.39^p^
	114-5	8.34 ± 0.12	8.23 ± 0.02	98.66 ± 0.19^def^	7.66 ± 0.01	91.85 ± 0.14^f^
	115-2	8.2 ± 0.22	7.55 ± 0.04	92.09 ± 0.44^n^	5.59 ± 0.09	68.17 ± 0.3^q^
	116-1	8.37 ± 0.07	8.27 ± 0.02	98.81 ± 0.18^cde^	6.36 ± 0.06	76.38 ± 0.12^n^
	116-2	8.43 ± 0.15	8.26 ± 0.02	98.05 ± 0.28^fgh^	7.64 ± 0.02	90.71 ± 0.26^g^
	117-3	8.39 ± 0.06	8.38 ± 0.01	99.87 ± 0.06^a^	8.00 ± 0.01	95.34 ± 0.12^c^
	119-2	8.52 ± 0.31	8.29 ± 0.03	97.30 ± 0.33^ij^	8.22 ± 0.01	96.6 ± 0.36^b^
	119-8	8.4 ± 0.26	8.33 ± 0.01	99.12 ± 0.13^bcde^	7.97 ± 0.01	94.87 ± 0.06^c^d
	120-4	7.86 ± 0.20	7.70 ± 0.03	98.00 ± 0.43^fghi^	6.65 ± 0.05	84.59 ± 0.11^j^
*L. plantarum*	032-1	8.42 ± 0.12	8.29 ± 0.01	98.44 ± 0.15^efg^	6.78 ± 0.03	80.55 ± 0.35^k^
	115-4	8.16 ± 0.36	8.11 ± 0.01	99.45 ± 0.12^abc^	7.89 ± 0.01	96.7 ± 0.09^a^
	117-5	7.75 ± 0.15	7.34 ± 0.05	94.73 ± 0.63^m^	7.30 ± 0.06	94.5 ± 0.26^d^
	118-10	8.18 ± 0.24	7.89 ± 0.03	96.52 ± 0.33^kl^	7.19 ± 0.02	87.98 ± 0.23^i^
	118-4	8.28 ± 0.19	8.02 ± 0.03	96.85 ± 0.4^jk^	7.36 ± 0.03	88.98 ± 0.41^h^
	118-9	8.26 ± 0.07	8.15 ± 0.05	98.65 ± 0.65^def^	7.75 ± 0.01	93.83 ± 0.06^e^
	119-1	8.31 ± 0.27	8.12 ± 0.03	97.71 ± 0.36^hi^	7.34 ± 0.02	88.48 ± 0.27^hi^
	119-5	8.27 ± 0.23	8.24 ± 0.01	99.55 ± 0.14^ab^	7.73 ± 0.02	93.46 ± 0.24^e^
	121-2	8.16 ± 0.22	9.13 ± 0.01	99.61 ± 0.15^ab^	8.1 ± 0.02	88.46 ± 0.18^hi^
	121-4	8.55 ± 0.13	8.36 ± 0.02	97.79 ± 0.22^ghi^	6.41 ± 0.04	75.01 ± 0.43^o^
	121-5	8.23 ± 0.08	8.14 ± 0.02	98.92 ± 0.2^bcde^	7.53 ± 0.03	91.46 ± 0.32^f^
	122-1	7.83 ± 0.36	7.75 ± 0.02	99.03 ± 0.31^bcde^	6.23 ± 0.06	79.56 ± 0.29^l^
	132-1	8.66 ± 0.32	7.65 ± 0.03	88.31 ± 0.36^o^	6.89 ± 0.02	79.5 ± 0.2^l^
	132-2	8.09 ± 0.29	8.02 ± 0.04	99.15 ± 0.57^bcd^	6.88 ± 0.01	84.97 ± 0.07^j^
	132-3	7.89 ± 0.21	6.87 ± 0.14	87.11 ± 0.42^p^	6.18 ± 0.03	78.36 ± 0.44^m^

*^a–z^Means in the same column with different lowercase letters differed significantly (P < 0.05).*

### Cell Surface Properties

**FIGURE 2:**
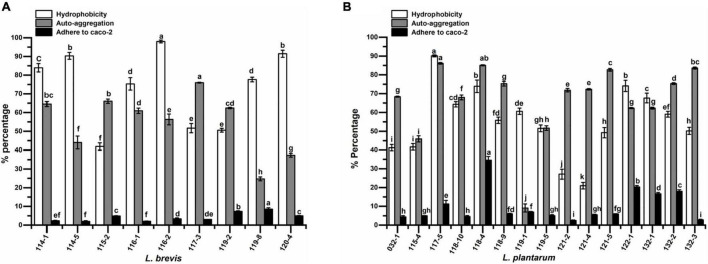
The hydrophobicity, auto-aggregation, and adhesion ability to Caco-2 cells of **(A)** *Lactobacillus brevis* strains and **(B)** *Lactobacillus plantarum* strains isolated from fermented vegetables in Shaanxi, China. Data shown are mean ± SD of triplicate values of independent experiments. Different letters indicate significantly different means (P < 0.05).

The isolates were tested for their cell surface hydrophobicity to estimate their adhesion ability by chloroform. Significant difference (*p* < 0.05) in hydrophobicity was found among different test strains. The five strains with the highest hydrophobicity were *L. plantarum* 117-5 with a value of 90.09%, and four strains of *L. brevis* (strains 116-2, 120-4, 114-5, and 114-1) with values of 98.03, 91.45, 90.31, and 83.94%, respectively. At the species level, the hydrophobicity values of *L. plantarum* ranged from 20.95 to 90.09% while those ranged from 41.87 to 98.03% for *L. brevis* (Figure 2).

Auto-aggregation of probiotic strains could affiliate adhesion of LAB to intestinal epithelium. The results of cell auto-aggregation assay are shown in Figure 2. The cell auto-aggregation rates of the isolates ranged from 24.73 to 75.98% (5 out of 9 above 60%) for *L. brevis* and 9.11 to 86.12% (12 out of 15 above 60%) for *L. plantarum*.

**FIGURE 3:**
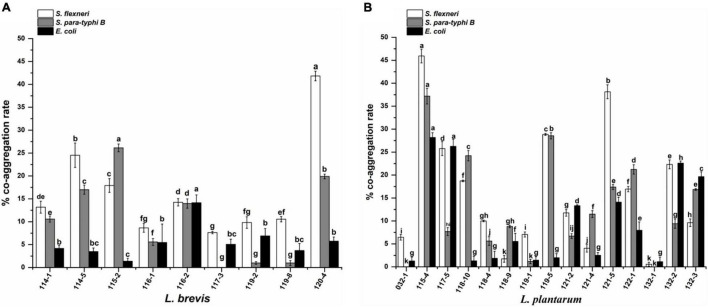
Co-aggregation of pathogenic bacteria with **(A)** *Lactobacillus brevis* strains and **(B)** *Lactobacillus plantarum* strains isolated from fermented vegetables in Shaanxi, China. The values represent the mean ± SD of three different assays. Different letters indicate significantly different means (*P* < 0.05).

The results of co-aggregation of *Lactobacillus* isolates in the presence of target pathogens are shown in Figure 3. The highest co-aggregation rates to *S. flexneri*, *S. paratyphi B*, and *E. coli* were obtained for isolate 115-4 (37.19, 45.93, and 28.19%, respectively). No obvious co-aggregation with all three pathogens was exhibited by *L. plantarum* 132-1 and 032-1. In addition, *L. brevis* 117-3, 119-2, and 119-8 showed no co-aggregation with *S. paratyphi B.*

### Adhesion to Caco-2

The adhesion capacity to human colon carcinoma cell line, Caco-2, was determined (Figure 2). Adhesion capacity to Caco-2 cells varied significantly among the tested *L. plantarum*, with adhesion ratio ranging from 2.42 to 34.52%. However, *L. brevis* showed adhesion capacity ranging from 1.98 to 8.53%. The top five strains with the highest adherence capacity all belonged to *L. plantarum*, namely isolates 118-4, 122-1, 132-2, 132-1, and 117-5, with mean values of 34.52, 20.27, 18.00, 16.69, and 11.26%, respectively.

### Antagonistic Activity of Lactic Acid Bacteria Isolates

The antagonistic activity against foodborne pathogenic bacteria displayed by the 24 selected *Lactobacillus* strains is shown in Table 3. In this experiment, after 18 h incubation, the *L. plantarum* strains showed significant antibacterial activity against all the enteric pathogens while *L. brevis* strains showed varied antagonistic activity. The pH values of *L. brevis* CFS were in the range of 4.31∼4.98, except for the strain 119-2 (pH 3.89), which showed the highest antagonistic activity among all *L. brevis* strains. The pH values of *L. plantarum* CFS were in the range of 3.64∼3.83. After pH neutralization to 6.5, all CFS showed minimal activity against all the pathogens tested, proving the role of organic acids for their antimicrobial activity.

**TABLE 3:** Antimicrobial activity of the cell-free supernatants of *Lactobacillus* strains from fermented vegetables in Shaanxi, China.

Species	Strains	pH of CFS	Zone of inhibition^1^		
			*Shigella flexneri*	*Salmonella paratyphi B*	*Escherichia coli*
*L. brevis*	114-1	4.76	–	++	+
	114-5	4.31	–	++	++
	115-2	4.87	–	–	–
	116-1	4.65	+	+	++
	116-2	4.81	–	+	+
	117-3	4.78	–	–	+
	119-2	3.89	+++	++	++
	119-8	4.98	–	+	+
	120-4	4.88	–	+	+
*L. plantarum*	032-1	3.69	+++	++	++
	115-4	3.66	+++	++	++
	117-5	3.71	+++	++	++
	118-10	3.68	+++	++	++
	118-4	3.83	++	++	+
	118-9	3.69	+++	++	++
	119-1	3.68	+++	++	++
	119-5	3.66	+++	++	++
	121-2	3.72	++	++	++
	121-4	3.74	+++	++	++
	121-5	3.77	+++	++	++
	122-1	3.7	++	++	++
	132-1	3.78	++	++	++
	132-2	3.64	++	++	++
	132-3	3.68	++	++	++

*^1^Results of independent experiments (n = 3) of inhibition zone (diameter in mm): –, no inhibition; +, 0∼3; ++, 3∼6; +++, >6. Diameter of well (8 mm) was deducted.*

### Antibiotic Susceptibility

The isolates were tested for their antibiotic susceptibility against different antibiotics (Table 4). Overall, all the isolates showed the ability to resist vancomycin, streptomycin, kanamycin, and ciprofloxacin while sensitive to ampicillin, cefixime, tetracycline, and erythromycin varied among strains, even species. All tested *L. plantarum* were susceptible to tetracycline while 3 out of 9 *L. brevis* strains were resistant to tetracycline. No resistance to erythromycin was found for both *L. brevis* (2M7S) and *L. plantarum* (4M11S). In addition, *L. brevis* showed more resistant toward ampicillin (2M7R) and cefixime (1M8R) than *L. plantarum*, which showed 1R1M13S toward ampicillin and 1R14S toward cefixime.

**TABLE 4:** Antibiotic susceptibility of *Lactobacillus* strains from fermented vegetables in Shaanxi, China.

Species	Strains	Streptomycin (10 μg/disk)	Kanamycin (30 μg/disk)	Vancomycin (30 μg/disk)	Ampicillin (10 μg/disk)	Cefixime (15 μg/disk)	Ciprofloxacin (5 μg/disk)	Tetracycline (30 μg/disk)	Erythromycin (15 μg/disk)
*L. brevis*	114-1	R	R	R	R	R	R	S	S
	114-5	R	R	R	R	R	R	S	S
	115-2	R	R	R	R	R	R	S	S
	116-1	R	R	R	R	R	R	S	S
	116-2	R	R	R	R	R	R	R	S
	117-3	R	R	R	R	R	R	R	S
	119-2	R	R	R	M	R	R	R	M
	119-8	R	R	R	M	R	R	S	M
	120-4	R	R	R	R	M	R	S	S
*L. plantarum*	032-1	R	R	R	S	S	R	S	S
	115-4	R	R	R	S	S	R	S	S
	117-5	R	R	R	S	S	R	S	S
	118-10	R	R	R	S	S	R	S	S
	118-4	R	R	R	M	R	R	S	M
	118-9	R	R	R	S	S	R	S	S
	119-1	R	R	R	S	S	R	S	S
	119-5	R	R	R	S	S	R	S	S
	121-2	R	R	R	S	S	R	S	S
	121-4	R	R	R	S	S	R	S	S
	121-5	R	R	R	S	S	R	S	S
	122-1	R	R	R	S	S	R	S	S
	132-1	R	R	R	R	S	R	S	M
	132-2	R	R	R	S	S	R	S	M
	132-3	R	R	R	S	S	M	S	M

*R, resistant; S, sensitive; M, moderately sensitive. The breakpoints for the antibiotic sensitivity/resistant in mm zone of inhibition: Ampicillin and vancomycin (≥17/≤14); Kanamycin (≥18/≤12); Tetracycline (≥19/≤14); Streptomycin (≥15/≤12); Cefixime (≥18/≤14); Ciprofloxacin (≥21/ ≤ 15); and Erythromycin (≥23/≤13).*

### Radical Scavenging Activity

DPPH and ABTS^+^ free radical scavenging experiments are commonly used to evaluate natural antioxidants (the antioxidant activity of LAB) (Sun et al., 2020).

**FIGURE 4:**
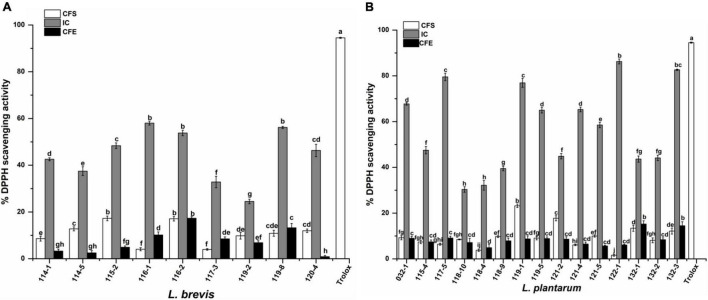
DPPH scavenging activity of **(A)** *Lactobacillus brevis* strains and **(B)** *Lactobacillus plantarum* strains isolated from fermented vegetables in Shaanxi, China. Data shown are mean ± SD of triplicate values of independent experiments. Different letters indicate significantly different means (*P* < 0.05).

As shown in Figure 4, the 24 strains tested showed varied degrees of radical scavenging activity. The intact cells of test strains showed the highest DPPH scavenging activity compared with corresponding CFS and CFE. The five highest scavenging activity (67.33, 79.54, 76.93, 86.29, and 82.66%) belonged to strains 032-1, 117-5, 119-1, 122-1, and 132-3, which were all *L. plantarum*. The lowest five values (37.46, 32.83, 24.51, 30.34, and 32.17%) belonged to *L. brevis* 114-5, 117-3, and 119-2, and *L. plantarum* 118-10 and 118-4.

**FIGURE 5:**
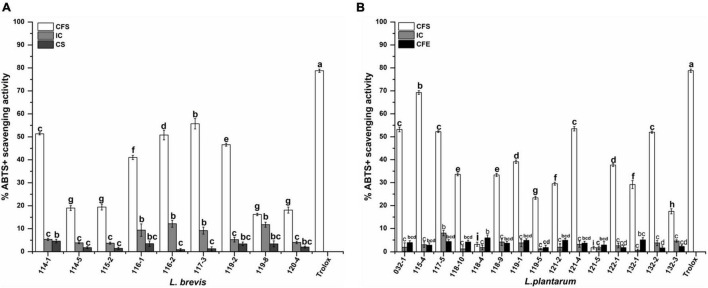
ABTS^+^ scavenging activity of **(A)** *Lactobacillus brevis* strains and **(B)** *Lactiplantibacillus plantarum* strains isolated from fermented vegetables in Shaanxi, China. Data shown are mean ± SD of triplicate values of independent experiments. Different letters indicate significantly different means (*P* < 0.05).

For the ABTS^+^ radical scavenging assay, as shown in Figure 5, the scavenging activity of CFS is significantly higher than IC and CFE. The highest scavenging value of CFS, IC, and CFE were obtained from strains 115-4 (69.26%), 116-2 (12.18%), and 118-4 (5.93%). The ABTS^+^ scavenging rate of CFS ranged from 16.22 to 55.70% for *L. brevis*. Interestingly, the CFS of *L. plantarum* 115-4 had the highest (69.26%) ABTS^+^ scavenging rate and that of *L. plantarum* 121-5 had the lowest rate (1.82%).

### α-Glucosidase Inhibitory Activity

**FIGURE 6:**
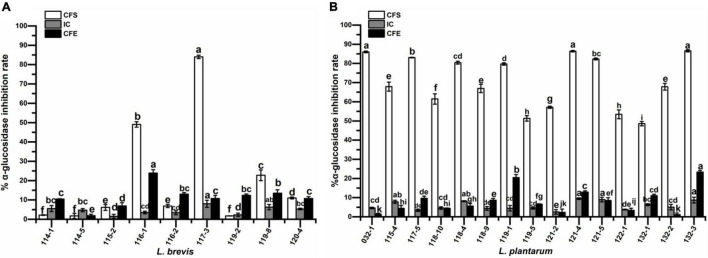
α-glucosidase inhibitory activity of **(A)** *Lactobacillus brevis* strains and **(B)** *Lactobacillus plantarum* strains isolated from fermented vegetables in Shaanxi, China. Data shown are mean ± SD of triplicate values of independent experiments. Different letters indicate significantly different means (*P* < 0.05).

The results of α-glucosidase inhibitory activities are shown in Figure 6. The inhibitory activities of CFS ranged from 1.74 to 84.01% for *L. brevis* while those of *L. plantarum* presented much higher values of 48.57–86.57%. The inhibitory activities of IC ranged from 2.55 to 9.45% for *L. plantarum*, which is similar to the corresponding CFE (between 1.13 and 23.34%) and much lower than the corresponding CFS. However, *L. brevis* exhibited similar levels for all the groups (IC: 1.47 to 8.01%; CFE: 1.87% ∼ 23.92%) except for the CFS of strain 117-3 and 116-1 with the much higher values of 84.01 and 49.06%, respectively. Out of 24 CFS of the isolates, 15, which all belonged to *L. plantarum* except for *L. brevis* 117-3, had inhibition rates higher than 50%.

### Cholesterol Assimilation

**FIGURE 7:**
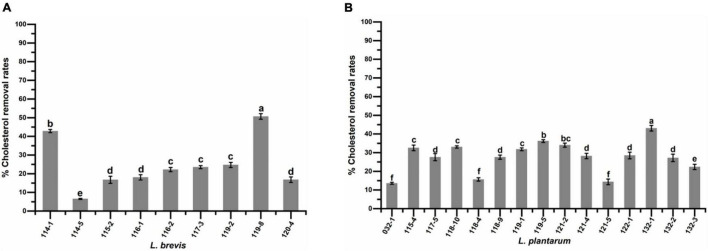
Cholesterol assimilation ability of **(A)** *Lactobacillus brevis* strains and **(B)** *Lactobacillus plantarum* strains isolated from fermented vegetables in Shaanxi, China. Data shown are mean ± SD of triplicate values of independent experiments. Different letters indicate significantly different means (*P* < 0.05).

Figure 7 presents the levels of cholesterol assimilation by isolates in the presence of 0.3% bile oxgall at 37°C for 24 h. The percentage of cholesterol assimilated varied (*P* < 0.05) in different strains and ranged from 6.53 to 50.64% for *L. brevis* and 13.59 to 43.06% for *L. plantarum*. The highest percentage of cholesterol assimilation was observed in isolates *L. brevis* 119-8 (50.64%), 114-1 (42.84%), *L. plantarum* 132-1 (43.06%), 119-5 (36.20%), and 121-2 (34.01%).

## Discussion

According to a previous study (Zielinska et al., 2015), LAB strains with vegetable origins are generally less bile-resistant and more low pH tolerance. Therefore, bile tolerance was used to prescreen the potential probiotics followed by pH tolerance test. The low pH tolerance test indicated that almost all isolates with high bile tolerance (exhibited below 50% suppression rate under 0.3% bile for 4 h) maintained growth under the pH condition as low as 2.0, which confirmed that LAB strains with vegetable origins had a high acid tolerance. Although *in vitro* assays under pH 3.0 has been preferred in most studies, survival of bacterial strains in low pH conditions is a more accurate indication of the ability of strains to survive passage through the stomach (Zielinska et al., 2015; Reuben et al., 2020).

In this study, 74 strains of potential LAB were isolated from fermented vegetables in Shaanxi, China. In total, 26 of 74 strains showed high bile tolerance and low pH tolerance and were identified based on 16S rRNA gene sequence. The result showed that the most abundant species was *L. plantarum* (15 strains) followed by *L. brevis* (9 strains). Similar to our result, *L. plantarum* and *L. brevis* were usually predominant in the different fermented vegetables, including Kimchi (Choi et al., 2018), Suancai and Paocai (Yu et al., 2012), Jiangshui (Zhang et al., 2018), De’ang pickled tea (Cao et al., 2019), and traditional fermented cabbage and cucumber (Zielinska et al., 2015). The dominance of *L. plantarum* in pickled vegetables has been attributed to its high acid tolerance (Tokatli et al., 2015; Zielinska et al., 2015). It is quite interesting that *Weissella viridescen* (2 strains), with high bile tolerance and low pH tolerance, were isolated in this study. It was reported that genus *Weissella* was one of the dominant bacteria in traditional fermented vegetables using DGGE analysis but no *Weissella* strain was isolated in the previous study (Zhang et al., 2018). The reason that *Weissella* strains were isolated may be that many samples were used in this study.

In addition to the effects of low pH and high bile salts on the survival rate of probiotics, tolerance to pepsin and pancreatic enzymes is also a key factor in the survival of probiotics once they enter the host. So, further investigation was conducted to assess the survival of LAB under the simulated gastrointestinal condition. All 24 selected *Lactobacillus* strains, after sequential exposure to simulated gastrointestinal juices, showed the survival rate higher than 68%. On a whole, the harsh environment tolerance of the isolates in this study was strain-specific, which was in accordance with the previous report (Tokatli et al., 2015).

The colonization in the intestinal wall is considered a desirable property of probiotic bacteria (Nami et al., 2019b). Hydrophobicity and auto-aggregation could contribute the colonization of LAB in the intestinal wall. In this study, all tested strains were positive for the auto-aggregation, hydrophobicity, and cell adherence to Caco-2 cells with varied levels. Out of 24 isolates, 9 exhibited both high cell surface hydrophobicity (>60%) and high auto-aggregation (>40%), which indicated their good colonization potential according to previous studies (Reuben et al., 2020). However, the adhesion to Caco-2 cell had no significant coherence to auto aggregation and hydrophobicity for the strains in this study. It was reported that adhesion of LAB is a multiplex phenomenon initiating with contact with host enterocytes followed by diverse surface interactions (Reuben et al., 2020).

Co-aggregation represents a defensive barrier for the colonization of pathogenic microorganisms (Choi et al., 2018; Nami et al., 2019b). The ability of LAB isolates to co-aggregate with pathogens could be attributed to proteinaceous components presented on the cell surface and interactions between carbohydrate and lectin (Nami et al., 2019b). In this study, foodborne pathogenic bacteria *S. flexneri, S. paratyphi B*, and *E. coli* were used and the results showed that co-aggregation ability depends on both *Lactobacillus* strains and pathogenic bacteria, which was in accordance with previous studies (Campana et al., 2017; Reuben et al., 2020). In addition, antagonistic activity test was conducted using the same foodborne pathogenic bacteria, and no correlation was observed between co-aggregation rate and antagonistic activity. The results also showed that *L. plantarum* strains exhibited significant antibacterial activity against all the enteric pathogens while varied antagonistic activity was exhibited by *L. brevis* strains. This phenomenon may be attributed to different organic acids production by the tested strains. It was reported that the increased production of organic acid through carbohydrates fermentation induced a pH decrease of the medium, which was a major factor suppressing the growth of the pathogen (Somashekaraiah et al., 2019).

Antibiotic susceptibility patterns are generally species-specific and geographical location of LAB is one of the factors that determine antibiotic susceptibility patterns of potential probiotic strains (Reuben et al., 2020). All tested *Lactobacillus* strains were resistant to streptomycin, kanamycin, vancomycin, and ciprofloxacin in this study. Resistance of vancomycin was considered to be intrinsic or natural resistances and therefore non-transmissible for *Lactobacillus* strains according to previous studies (Hummel et al., 2007; Zielinska et al., 2015; Cao et al., 2019). The intrinsic resistance of probiotic strains promotes both therapeutic and preventive benefits when administered together with antibiotics, as intestinal microbiota recovery becomes facilitated (Das et al., 2020; Reuben et al., 2020). There are also reports suggesting that transfer of streptomycin, kanamycin, and ciprofloxacin resistance is still lacking in *Lactobacillus* (Das et al., 2020). In addition, resistances to tetracycline, ampicillin, and cefixime were exhibited by several strains, which were consistent with previous findings (Somashekaraiah et al., 2019; Reuben et al., 2020), congruent with most commercial probiotics (Sharma et al., 2014). However, it was found that LAB strains may harbor resistance genes which may be transferred to pathogenic bacteria (Hummel et al., 2007; Nawaz et al., 2011; Campedelli et al., 2019). Therefore, their transferability to other genera requires further in-depth study.

*In vitro* studies on cholesterol reduction, α-glucosidase inhibition, and radical scavenging activities have been considered as important parameters for the selection of probiotic strains with diverse health-promoting benefits.

Reactive oxygen species (ROS) are generated in the human body by different endogenous systems under various conditions. Excess free radicals in the human system adversely affect macromolecules including lipids, proteins, and deoxyribonucleic acid (DNA), leading to aging, arthritis, cardiovascular diseases, cancer, diabetes, and neurodegenerative diseases (Ragul et al., 2020). Due to the limitation of a single antioxidant property test to reflect the antioxidant capacity of the isolates, both ABTS^+^ and DPPH radical scavenging capacities were used to investigate the antioxidant activities of probiotic strains (Cao et al., 2019; Ragul et al., 2020). This study showed that both the DPPH and ATBS^+^ scavenging rate varied significantly for different *Lactobacillus* strains, which was in accordance with the previous studies (Chen et al., 2014; Ragul et al., 2020). Interestingly, the DPPH scavenging rate of IC was higher than those of the corresponding CFE and CFS for almost all strains while the highest ATBS^+^ activities were exhibited by CFS in this study. Our result was consistent with previous reports (Chen et al., 2014). However, it was reported that both the DPPH and ABTS^+^ scavenging rates of CFS were higher than those of the IC and CFE for LAB strains (Cao et al., 2019). We speculated that radical scavenging rate was significantly affected by the strain origin.

α-Glucosidase, which is in the brush border membrane, catalyzes the digestive process of carbohydrates. Inhibiting α-glucosidase activity has been demonstrated to decrease glucose absorption and reduce blood glucose levels (Chen et al., 2014). In this study, inhibitory activity on α-glucosidase was observed for all strains with varied values. Similar results were also obtained by previous studies (Chen et al., 2014). Interestingly, CFS of *L. plantarum* strains exhibited much higher inhibition rates than those of most *L. brevis* strains, which could be speculated that different activity substances were secreted by these two species. In addition, CFS of seven strains showed potent α-glucosidase inhibition rate with the values above 80%, indicating their potential as promising α-glucosidase inhibitor.

High concentration of cholesterol in the bloodstreams of humans is generally recognized as a risk factor for coronary heart disease (Tokatli et al., 2015). Consumption of fermented products containing certain *lactobacilli* has been shown to reduce serum cholesterol levels in humans (Tokatli et al., 2015; Li et al., 2019). In this study, all the strains showed cholesterol assimilation ability but with varied values, which was in accordance with the previous study (Tokatli et al., 2015). The highest cholesterol assimilation rate was 50.64%, corresponding to the amount of approximately 50 μg/mL, which was higher than those of *Lactobacillus* strains obtained from Chinese traditional sourdough (20∼30 μg/mL) (Li et al., 2019) and traditional cubuk pickles (20 μg/mL) (Tokatli et al., 2015), but lower than those (62.63 μg/mL) of *Lactobacillus* strains obtained from Tibetan kefir grains (Zheng et al., 2013). Previous studies also indicated *lactobacilli* strains with cholesterol assimilation ability *in vitro* could reduce cholesterol level *in vivo* (Zheng et al., 2013; Nami et al., 2019a).

It is well-known that the probiotic properties of *Lactobacillus* spp. were strain-specific (Ramasamy et al., 2012; Campana et al., 2017; Somashekaraiah et al., 2019) and few comparative studies among different species of *Lactobacillus* with the same origin have been conducted (Ramos et al., 2013; Tokatli et al., 2015). In this study, it was first found that *L. plantarum* generally had higher antibacterial activities, α-glucosidase inhibition ability, and antibiotics susceptibility compared to *L. brevis*, which might provide valued reference for future screening of probiotics. In addition, it is worthy to notice that the specific probiotic properties with the highest values were exhibited by different strains, which was a coincidence with previous reports and thus several strategies were proposed to select the best probiotic (Mallappa et al., 2019; Nami et al., 2020). In our opinion, potential probiotics should be selected based on the specific purpose. Anyway, further evaluation *in vivo* would be needed to obtain more information on probiotic potential of the isolates.

## Conclusion

In this study, probiotic potential of *Lactobacillus* isolated from fermented vegetables in Shaanxi, China, was first evaluated *in vitro*. The results showed that the probiotic characteristics were strain-dependent, and several strains exhibited excellent probiotic potential based on their survival rate to transit simulated gastrointestinal tract, cell surface hydrophobicity, auto-aggregation, co-aggregation with pathogen, adhesion to Caco-2, antimicrobial activity, antibiotics susceptibility, radical scavenging ability, α-glucosidase inhibition, and the cholesterol assimilation. Interestingly, it was first found that *L. plantarum* generally had higher antibacterial activities, α-glucosidase inhibition, and antibiotics susceptibility compared to *L. brevis*. On the whole, results obtained in this study indicated that the *Lactobacillus* strains from handmade fermented vegetables in Shaanxi province, China, could be exploited as a promising source of novel probiotic LAB.

## Data Availability Statement

The datasets presented in this study can be found in online repositories. The names of the repository/repositories and accession number(s) can be found in the article/Supplementary Material.

## Author Contributions

CL did the experiments and performed the data analysis, with help from W-JX and CA. HD collected fermented vegetable samples for the experiment. W-JX supervised the project. S-JM and YL revised the manuscript. All authors have contributed to the intellectual input and assistance to this study and to manuscript preparation, read and approved the final manuscript.

## Conflict of Interest

The authors declare that the research was conducted in the absence of any commercial or financial relationships that could be construed as a potential conflict of interest.

## Publisher’s Note

All claims expressed in this article are solely those of the authors and do not necessarily represent those of their affiliated organizations, or those of the publisher, the editors and the reviewers. Any product that may be evaluated in this article, or claim that may be made by its manufacturer, is not guaranteed or endorsed by the publisher.
